# Complete Genome of *Avian coronavirus* Vaccine Strains Ma5 and BR-I

**DOI:** 10.1128/genomeA.00201-17

**Published:** 2017-06-08

**Authors:** Paulo E. Brandão, Sueli A. Taniwaki, Mikael Berg, Aline S. Hora

**Affiliations:** a Department of Preventive Veterinary Medicine and Animal Health, School of Veterinary Medicine, University of São Paulo, São Paulo, Brazil; b Section of Virology, Department of Biomedical Sciences and Veterinary Public Health, Swedish University of Agricultural Sciences (SLU), Uppsala, Sweden

## Abstract

*Avian coronavirus* (AvCoV) is a ubiquitous multiple-serotype pathogen of poultry, and its control is mainly based on the use of vaccines. We report here the previously unknown full genomes of the Ma5 (27,652 nucleotides [nt]) and BR-I (27,618 nt) AvCoV vaccine strains of the GI-1 (Massachusetts) and GI-11 (Brazil) types.

## GENOME ANNOUNCEMENT

Avian infectious bronchitis virus, ubiquitously found in and highly infectious to chickens, is currently classified as *Avian coronavirus* (AvCoV; *Nidovirales*:*Coronaviridae*:*Coronavirinae*:*Gammacoronavirus*) with an approximately 27-kb positive-sense, single-stranded, 5′-capped genomic RNA. This genome is organized as 5′ untranslated region (UTR)/open reading frame (ORF) 1ab (replicase polyprotein)/receptor-binding spike S glycoprotein/envelope E protein/membrane M protein /nucleocapsid N protein /3′ UTR, with accessory proteins 3a and 3b (upstream of the E protein gene) and 5a and 5b (upstream of the N protein gene) (1, 2).

A variety of vaccine strains derived from the hitherto described 6 genotypes/32 lineages have been used to control the disease (3, 4). The availability of full genomes for vaccine strains allows for the search for genetic markers of attenuation, protection, and mutation, as well as for comparison with field strains. Here, we report the previously unavailable full genomes of strains Ma5 (GI-1, Massachusetts type) and BR-I (GI-11, Brazil type).

Total RNA extraction with TRIzol Reagent (Life Technologies, Inc.) and an RNeasy mini kit (Qiagen) was carried out on commercially available live vaccines with each of the two strains after the samples were filtered (0.45 µm) and treated with DNase/RNase; random double-stranded cDNAs were synthesized using Superscript III and Klenow exo-DNA polymerase (Life Technologies, Inc.). Libraries were constructed using Illumina kits (Nextera XT Index and Nextera XT DNA), and paired-end reads were sequenced with a NextSeq500 instrument (Illumina) using the NextSeq500 version 2 high-output sequencing kit (300 cycles). The consensus sequences were assembled with CLC Genomics Workbench version 9.5.3 (Qiagen) with AvCoV sequences FJ888351.1 and KX258195.1 as references for Ma5 and BR-I, respectively, and with a read-trimming cutoff of 0.05. A total of 57,827,656 and 132,767,024 paired-end sequences were obtained, from which 9,382,185 and 93,704,684 were mapped for Ma5 and BR-I, respectively.

The resulting genomes for Ma5/BR-I were 27,652/27,618 nucleotides (nt) long, including the poly(A) tails, and organized as 5′ UTR (nt 1 to 528/1 to 528), ORF1a (nt 529 to 12324/529 to 12324), and ORF1ab (nt 529 to 20363/529 to 20363), with a ribosomal frameshift between these two ORFs, followed by the spike gene S (nt 20314 to 23802/20314 to 23823), 3a (nt 23802 to 23975/23823 to 23996), 3b (nt 23975 to 24169/23996 to 24187), envelope E (nt 24150 to 24479/24171 to 24494), membrane M (nt 24451 to 25128/24463 to 25143), 5a (nt 25488 to 25685/25503 to 25700), 5b (nt 25682 to 25930/25697 to 25945), and nucleocapsid N (nt 25873 to 27102/25888 to 27114), with a 3′ UTR at nt 27103 to 27652/27115 to 27618, including the poly(A) tails.

No recombination was found for the Ma5 and BR-I genomes using a set of AvCoV genomes in RDP4 (5). Nucleotide identities between Ma5 and BR-I were 90.5% for the full genomes and 82.6% for the spike gene. The mean Jameson-Wolf antigenic index (6) for the spike proteins of both viruses was 0.39, though at site 38, to which a role in tropism has been described (7), the index was 2.52 for Ma5 (Asp) and 0.2 for BR-I (Asn).

Because vaccination is a core tool for controlling AvCoV infection and due to the range of variables to be considered for this process (8), the integration of experimental, field, and genomic data could improve the process of developing vaccines and predicting their behavior.

### Accession number(s).

The complete genomes of strains BR-I and Ma5 have been deposited in GenBank under the accession numbers KY626044 and KY626045, respectively.
